# The Global Sink of Available Potential Energy by Mesoscale Air‐Sea Interaction

**DOI:** 10.1029/2020MS002118

**Published:** 2020-10-21

**Authors:** Stuart P. Bishop, R. Justin Small, Frank O. Bryan

**Affiliations:** ^1^ Department of Marine, Earth, & Atmospheric Sciences North Carolina State University at Raleigh Raleigh NC USA; ^2^ National Center for Atmospheric Research Boulder CO USA

## Abstract

The thermal component of oceanic eddy available potential energy (EPE) generation due to air‐sea interaction is proportional to the product of anomalous sea surface temperature (SST) and net air‐sea heat flux (SHF). In this study we assess EPE generation and its timescale and space‐scale dependence from observations and a high‐resolution coupled climate model. A dichotomy exists in the literature with respect to the sign of this term, that is, whether it is a source or a sink of EPE. We resolve this dichotomy by partitioning the SST and net heat flux into climatological mean, climatological seasonal cycle, and remaining transient contributions, thereby separating the mesoscale eddy variability from the forced seasonal cycle. In this decomposition the mesoscale air‐sea SST‐SHF feedbacks act as a 0.1 TW global sink of EPE. In regions of the ocean with a large seasonal cycle, for example, midlatitudes of the Northern Hemisphere, the EPE generation by the forced seasonal cycle exceeds the mesoscale variability sink, such that the global generation by seasonal plus eddy variability acts as a 0.8 TW source. EPE destruction is largest in the midlatitude western boundary currents due to mesoscale air‐sea interaction and in the tropical Pacific where SST variability is due mainly to the El Niño–Southern Oscillation. The EPE sink in western boundary currents is spatially aligned with SST gradients and offset to the poleward side of currents, while the mean and seasonal generation are aligned with the warm core of the current. By successively smoothing the data in space and time we find that half of the EPE sink is confined to timescales less than annual and length scales less than 2°, within the oceanic mesoscale band.

## Key Points

- Air‐sea interaction at scales less than 2° and annual accounts for half of the global destruction of EPE
- The forced mean seasonal cycle is a source of available potential energy
- Mean and transient energy sinks are spatially offset and colocated with mean SSH and SST gradients, respectively

## 1 Introduction

The turbulent ocean circulation is replete with mesoscale eddies, transient features that vary on O(10–100 km) spatial scales and monthly and longer timescales (Chelton et al., 2011). They are commonly observed as cyclonic and anticylonic vorticies but can also appear as waves and meanders propagating in major ocean currents such as the western boundary currents. The surface expressions of these eddies are apparent in multiple decades of remotely sensed sea surface temperature (SST), height (SSH), and wind (i.e., QuickSCAT). The impact of mesoscale SST variability on the surface wind has drawn considerable attention in the recent literature (Chelton et al., 2004), while its relationship to turbulent surface heat fluxes has received less attention. This can be attributed at least in part to the paucity of high‐resolution observations of heat flux, a derived quantity that requires colocated and contemporaneous observations of both near‐surface atmosphere and ocean state variables (Fairall et al., 2003).

Great advances in ocean modeling over the past couple decades now permit the explicit simulation of mesoscale eddies in coupled climate models. Early modeling studies that did not resolve mesoscale eddies, but instead parameterized their impacts on the ocean circulation (Gent & McWilliams, 1990) (hereafter referred to as GM), showed that the ocean's SST variability is primarily a passive response to atmospheric forcing (Frankignoul & Kestenare, 2002). More recently, coupled model simulations that explicitly resolve mesoscale eddies and concurrent advances in observations of the global turbulent surface heat flux have both revealed a more complex picture in which SST is not simply a passive response to atmospheric forcing (Bishop et al., 2017; Kirtman et al., 2012; Penduff et al., 2011, 2018; Small et al., 2019, 2020). Rather, in regions of the ocean with strong currents, SST variability is governed more by anomalous advection associated with variability arising from intrinsic oceanic processes (Roberts et al., 2017; Small et al., 2020). It is also becoming more evident that resolving mesoscale features can impact the synoptic weather band (X. Zhang et al., 2019). This points to a significant need to incorporate understanding of these air‐sea feedback processes into mesoscale eddy subgrid parameterizations that will continue be required in coarse‐resolution Earth system models for possibly decades to come.

The GM parameterization was developed to mimic baroclinic instability and serves as a net sink of available potential energy (APE) in the interior of the ocean under adiabatic conditions. Recent efforts to improve mesoscale eddy parameterizations have focused on a more comprehensive accounting for the eddy energy cycle (Zanna et al., 2020). Mesoscale air‐sea feedbacks are a potential diabatic source or sink of energy and thus will need to be considered in these newer frameworks. However, there are conflicting studies in the literature that show that mesoscale air‐sea feedbacks, which are proportional to the product of SST and air‐sea net heat flux anomalies, can be a source of APE (von Storch et al., 2012) or a sink (Ma et al., 2016; Shan et al., 2020; Yang et al., 2019). The ambiguity may arise in part from differing definitions of “eddy” and the region under consideration. The former (von Storch et al., 2012) is a global analysis and includes all transient variability including the seasonal cycle in their definition of “eddy.” The latter (Ma et al., 2016; Shan et al., 2020; Yang et al., 2019) are focused on the Kuroshio Extension system in the North Pacific and focus exclusively on the winter season. All of the cited studies are model based. Since global coupled models often result in elevated SST variance compared with observations (Small et al., 2014), there remains considerable uncertainty in how strong of an impact this source or sink of APE is.

In this study we seek to establish two objectives. We first will clarify the nature of mesoscale air‐sea interaction as either a global source or sink of eddy available potential energy (EPE) using state‐of‐the‐art observational SST and net surface heat flux products and compare these estimates against a global high‐resolution coupled climate simulation. We next determine the relevant timescales and space scales of the mesoscale air‐sea interaction that contribute to EPE generation and destruction. By doing so we will build a base upon which to constrain future model‐based analyses and parameterization development.

## 2 Methods

### 2.1 Available Potential Energy Generation

Following von Storch et al. (2012), the generation of APE is the surface area ( A) integral of the product of surface buoyancy anomalies from a reference state (*b*_∗_) and air‐sea buoyancy fluxes ( $B_{o}$),

(1) $$G_{a}=\int_{A}\rho_{o}\frac{\bar{b_{*}B_{o}}}{N_{r}^{2}}dA.$$

In (1) *ρ*_*o*_ is a constant reference density for a Boussinesq fluid, *b*_∗_ = *b* − *b*_*r*_(*z*), where *b* is buoyancy, *b*_*r*_(*z*) is a reference buoyancy, and $N_{r}^{2}=db_{r}/dz$ is the reference buoyancy frequency. The over bar, $\bar{\left(\;\right)}$, is a time average. The air‐sea buoyancy flux is a combination of net heat flux (*Q*_*o*_) and freshwater fluxes,

(2) $$B_{o}=\frac{\alpha_{\theta }g}{\rho_{o}c_{p}}Q_{o}-\frac{\alpha_{S}g}{\rho_{fw}}S(E-P),$$

where *α*_*θ*_ is the thermal expansion coefficient, *c*_*p*_ is the specific heat at constant pressure, *α*_*S*_ is the salinity contraction coefficient, *ρ*_*fw*_ is freshwater density, *S* is the surface salinity, and *E* − *P* is evaporation minus precipitation encompassing the freshwater fluxes.

In this study we will only consider the thermal component of the generation of APE. Using a simple equation of state for seawater, buoyancy can be expressed as *b*_∗_ = *α*_*θ*_*gθ*_∗_ = *α*_*θ*_*g*[*θ* − *θ*_*r*_(*z*)], and we can approximate (1) as

(3) $$G_{a}\approx \int_{A}\frac{\alpha_{\theta }^{2}g^{2}}{c_{p}N_{r}^{2}}\bar{\theta_{*}Q_{o}}dA.$$

The sign convention used for net heat flux is positive into the ocean. When (3) is a source of APE it is positive and occurs when cool SST corresponds with net heat flux out of the ocean and vice versa for warm SSTs.

### 2.2 Three‐Way Decomposition of APE Generation

As a proxy for the total generation of APE per unit volume we will examine the time‐averaged product of the SST anomaly *θ*_∗_ from a reference state *θ*_*r*_(*z*) and net air‐sea heat flux (SHF) *Q*_*o*_. We further decompose the product into a climatological time mean, a mean seasonal cycle, and a transient eddy contribution,

(4) $$\bar{\theta_{*}Q_{o}}=\theta_{*}^{m}Q_{o}^{m}+\bar{\theta_{*}^{s}Q_{o}^{s}}+\bar{\theta_{*}^{\prime }Q_{o}^{\prime }},$$

where the *m* and *s* superscripts indicate the time‐mean and seasonal climatology anomaly, respectively. See Appendix A for a full derivation of (4); (4) multiplied by $\alpha_{\theta }^{2}g^{2}/c_{p}N_{r}^{2}$ is proportional to the rate of APE generation in units of W m^−2^. Scaling in the midlatitudes shows that 10^2^°C W m^−2^ is approximately 1 mW m^−2^ in APE units. This decomposition follows Bryan et al. (2014) and Griffies et al. (2015), which define an eddy as a deviation from the monthly mean climatology and differs from von Storch et al. (2012), which includes a mean seasonal cycle embedded in the transient eddy term. The time mean as a mean state will be referred to as the traditional approach. Many of the pioneering works on ocean energetics that used relatively short temporal records from moored current meter moorings in the Southern Ocean and Gulf Stream used ensemble time averages as the mean state to estimate EPE and EKE budgets (Bryden, 1979; Cronin & Watts, 1996; Dewar & Bane, 1989). The work here highlights the importance of isolating the mean seasonal cycle when doing an eddy‐mean decomposition when using surface data and or long temporal records that can resolve a robust seasonal cycle.

The first, second, and third terms on the right‐hand side of (4) will be referred to as the local mean, seasonal, and transient APE generation terms, respectively. The global rate of thermal APE generation by the mean (*G*_*m*_), seasonal (*G*_*s*_), and transient (*G*_*e*_) SST and net air‐sea heat flux can be written by integrating (4) over the surface area ( A) of the ocean,

(5) $$G_{m}=\int_{A}\frac{\alpha_{\theta }^{2}g^{2}}{c_{p}N_{r}^{2}}\theta_{*}^{m}Q_{o}^{m}dA,$$

(6) $$G_{s}=\int_{A}\frac{\alpha_{\theta }^{2}g^{2}}{c_{p}N_{r}^{2}}\bar{\theta_{*}^{s}Q_{o}^{s}}dA,$$

(7) $$G_{e}=\int_{A}\frac{\alpha_{\theta }^{2}g^{2}}{c_{p}N_{r}^{2}}\bar{\theta_{*}^{\prime }Q_{o}^{\prime }}dA.$$

From a Lorenz energy cycle (Lorenz, 1955) standpoint, the mean (5) and seasonal (6) terms play a role in the mean APE reservoir, while the transient eddy (7) is a source or sink of EPE. In section 3, we will examine the integrands in (5)–(7) as well as partial (zonal) and full (global) integrals.

### 2.3 Data

#### 2.3.1 Observations

The observational data sets used in this study will primarily be SST and net air‐sea heat flux with a combination of mean subsurface climatology. The net heat flux is a combination of shortwave, longwave, and turbulent latent and sensible heat fluxes from the Japanese Ocean Flux data sets with Use of Remote sensing Observations Version 3 (J‐OFURO3). J‐OFURO3 is the evolution of the original J‐OFURO data set (Kuboto et al., 2002; Tomita et al., 2010). The new version is available for 1988–2013 with daily and monthly mean temporal resolution and 0.25° spatial resolution. The focus of this study is on the monthly mean product, which is a complete data set compared with the daily product that has data gaps between satellite orbiting tracks. J‐OFURO3 is derived solely from satellite data except for 2 m air temperature taken from NCEP‐DOE reanalysis (Kanamitsu et al., 2002). Daily averaged SST is an ensemble median of multiple satellite data sources and of Reynolds et al. (2007) SST. The fluxes are computed using the COARE 3.0 bulk flux algorithm (Fairall et al., 2003). We use 20 years of data from 1993–2013, which coincide with years in which altimetric SSH data are available. Full details of the data set are given in Tomita et al. (2019). The J‐OFURO3 data set was found to give consistent results for SST‐SHF covariance with other flux data sets in Small et al. (2020), but it is acknowledged that considerable uncertainty exists between products for other metrics such as heat flux variance and trend (R. Zhang et al., 2018). The surface reference temperature used in this study is the time‐ and surface area‐averaged SST from J‐OFURO3, *θ*_*r*_(*z* = 0) = 13.5°C.

The Monthly Isopycnal and Mixed‐layer Ocean Climatology (MIMOC) (Schmidtko et al., 2013) is an Argo float derived hydrographic climatology with 1° resolution and is used to provide the mean mixed layer depth ( $\bar{h}$) and estimate the spatial distribution of the mean surface thermal expansion coefficient, ${\bar{\alpha }}_{\theta }(x,y)$, in order to convert SST to buoyancy from the simple linear EOS. The mixed layer depth and thermal expansion coefficients were remapped to the 0.25° J‐OFURO3 grid through linear interpolation. ${\bar{\alpha }}_{\theta }(x,y)$ ranges from 1–5 × 10^−4^°C^−1^ with higher values in warmer regions of the ocean in the tropics and midlatitudes and lower values in the subpolar oceans. MIMOC was also used to estimate the background reference buoyancy frequency as the time‐ and volume‐averaged buoyancy frequency, *N*_*r*_ = 5.2 × 10^−3^ s ^−1^. Other values needed to compute (7) are the reference density, *ρ*_*o*_, here taken to be 1,025 kg m^−3^, specific heat at constant pressure, *c*_*p*_ ≈ 4,000 J kg^−1^ °C^−1^, and gravitational acceleration, *g*, taken to be a constant 9.81 m s^−2^. For consistency and comparing between observations and models the observational values for the reference SST and buoyancy frequency *N*_*r*_ will be used in the analysis of the model output. This only matters for estimates of *G*_*m*_ (Equation 5 used as a metric), which cancels out for the seasonal and transient eddy contributions. Since we are not trying to fully close the total energy budget, the magnitude of the APE is somewhat arbitrary. A more accurate depiction of the reference state would need full water column stratification to determine the reference state for APE; refer to Molemaker and McWilliams (2010) for more sophisticated methods.

AVISO (Ducet & Le‐Traon, 2001) provides a 0.25° resolution SSH anomaly monthly averaged product that will be used to calculate mixed layer depth EKE (11) and assess the colocation of energy generation features to the mean paths of the major ocean currents. The AVISO grid is the same as the J‐OFURO3 grid, and we use the same time frame 1993–2013.

#### 2.3.2 Mesoscale Eddy‐Resolving Coupled Climate Model

The model used is a high‐resolution version of the Community Earth System Model (Hurrell et al., 2013), which will be referred to as CESM‐H. It is a climate system model that is the successor to the Community Climate System Model, Version 4 (Gent et al., 2011). Details of the simulation examined are summarized below, but for a more in‐depth description, see Small et al. (2014). The model configuration includes the Community Atmosphere Model, Version 5 (CAM5), with a spectral element dynamical core; Community Ice Code, Version 4 (Hunke & Lipscomb, 2008); Parallel Ocean Program, Version 2; and Community Land Model, Version 4 (Lawrence et al., 2011). CAM5 was integrated with a horizontal resolution of about 0.25° (specifically, the spectral element dynamical core with 120 elements on each face of the cubed sphere, referred to as ne120) and 30 levels in the vertical.

The POP2 model has a nominal grid spacing of 0.1° (decreasing from 11 km at the equator to 2.5 km at high latitudes) on a tripole grid with poles in North America and Asia. The configuration is similar to that used in McClean et al. (2011) and Kirtman et al. (2012), except that the number of vertical levels was increased from 42 to 62, with more levels in the main thermocline. The ocean communicated with the coupler, providing updated SST and surface currents and receiving updated surface fluxes, every 6 hr, and the atmosphere communicated every 10 min. The coupler computes air‐sea fluxes using the Large and Yeager (2009) surface layer scheme. The land and sea ice models are on the same grids as the atmosphere and ocean models, respectively. POP2 has been shown to produce eddy transports consistent with mooring‐based observations in the Kuroshio Extension region (Bishop & Bryan, 2013) and Southern Ocean (Lenn et al., 2011). Abernathey and Wortham (2015) show that it also reproduces the spectral and cospectral characteristics of near‐surface geostrophic velocity and SST observed via remote sensing.

For this study we will use model years 60–95 of the 100 year run providing 35 years of output to compare with 21 years of observations. In order to provide consistency between observations and CESM‐H we use monthly averaged output, and additionally, the fields were remapped to the 0.25° observational grid using the NCL/ESMF routines with bilinear remapping.

### 2.4 Removing El Niño–Southern Oscillation

One of the findings of this study is that *G*_*e*_ in the tropics is dominated by variability related to the El Niño–Southern Oscillation (ENSO). In order to assess the importance of the ENSO cycle on the globally integrated transient EPE generation, we follow the methods of Bishop et al. (2017) to remove a major portion of the ENSO signal by removing the linear regression of the Niño 3.4 index. The Niño 3.4 index, $\theta_{34}^{\prime }(t)$, is defined as the area‐averaged SST anomaly between 120–170°W and 5°S to 5°N. This index is representative of the first mode of the EOF method outlined in Frankignoul et al. (2011), which represents 61% of the variance. We define the component of the transient SST variability associated with ENSO as

(8) $$\theta_{enso}^{\prime }(x,y,t)=m(x,y)\theta_{34}^{\prime }(t)+b(x,y),$$

where *m*(*x*,*y*) is the slope of the *θ′*(*x*,*y*,*t*) versus $\theta_{34}^{\prime }(t)$ scatterplot and *b*(*x*,*y*) is the *y* intercept. The modified SST anomaly ( $\theta_{no34}^{\prime }$) is used to estimate (7) with ENSO removed,

(9) $$\theta_{no34}^{\prime }(x,y,t)=\theta^{\prime }(x,y,t)-\theta_{enso}^{\prime }(x,y,t).$$

This method allows us to determine how much of the large‐scale ENSO signal is contributing to energetics in the tropics and whether there are any instantaneous ocean teleconnections to the midlatitude oceans.

### 2.5 Scale Dependence

To assess the scale dependence of EPE generation and destruction we evaluate the integrand of (7) with anomaly fields spatially and temporally smoothed to varying degrees. The anomaly SST and net heat flux fields were spatially smoothed in latitude and longitude from 0.5–10° by 0.5° increments using a 2‐D boxcar filter (implemented in the convolution module of Python astropy (https://www.astropy.org/). The anomaly fields were low‐pass filtered in time at each grid point using a fourth‐order Butterworth filter and successively increasing the cutoff frequency from 3 months to 5 years by 3 month increments.

## 3 Results

### 3.1 Mixed Layer Eddy Energetics

Within the constraints of the remote sensing observations we employ in this investigation, the global EPE in the mixed layer is approximated by assuming that the SST is constant throughout the mixed layer. This was confirmed in midlatitudes at the Kuroshio Extension Observatory (Cronin et al., 2013). The volume‐integrated mixed layer EPE (with the origin of the vertical coordinate taken at the sea surface) is approximately

(10) $$P_{e}=\int_{-\bar{h}}^{0}\int_{A}\frac{\rho_{o}}{2}\bar{\frac{b_{*}^{\prime 2}}{N_{r}^{2}}}dAdz\approx \int_{A}\frac{\rho_{o}}{2}\frac{\alpha_{\theta }^{2}g^{2}}{N_{r}^{2}}\bar{\theta_{*}^{\prime 2}}\;\bar{h}dA,$$

where $\bar{h}(x,y)$ is the mean mixed layer depth and salinity variations are neglected. Similarly, an upper bound on the volume‐integrated EKE is approximately

(11) $$K_{e}=\int_{-\bar{h}}^{0}\int_{A}\frac{1}{2}\rho_{o}(\bar{{u^{\prime }}^{2}}+\bar{{v^{\prime }}^{2}})dAdz\approx \int_{A}\frac{1}{2}\rho_{o}(\bar{{u^{\prime }}^{2}}+\bar{{v^{\prime }}^{2}})\bar{h}dA,$$

where *u* and *v* are the surface geostrophic zonal and meridional velocities, respectively, calculated from the SSH anomaly (*η′*), $u^{\prime }=g/f\hat{k}\times ▽\eta^{\prime }$ and $\hat{k}$ is the vertical unit vector; (11) is an upper bound because it is assumed that the surface geostrophic velocities are uniform in the vertical throughout the mixed layer. EKE was not computed within the tropics between ±10° latitude. The vertical structure of the near‐surface flow within frontal systems such as midlatitude western boundary currents is likely in turbulent thermal wind balance (McWilliams et al., 2015; Wenegrat et al., 2018), but that level of detail is not accessible with remote sense‐based observations.

**Figure 1:**
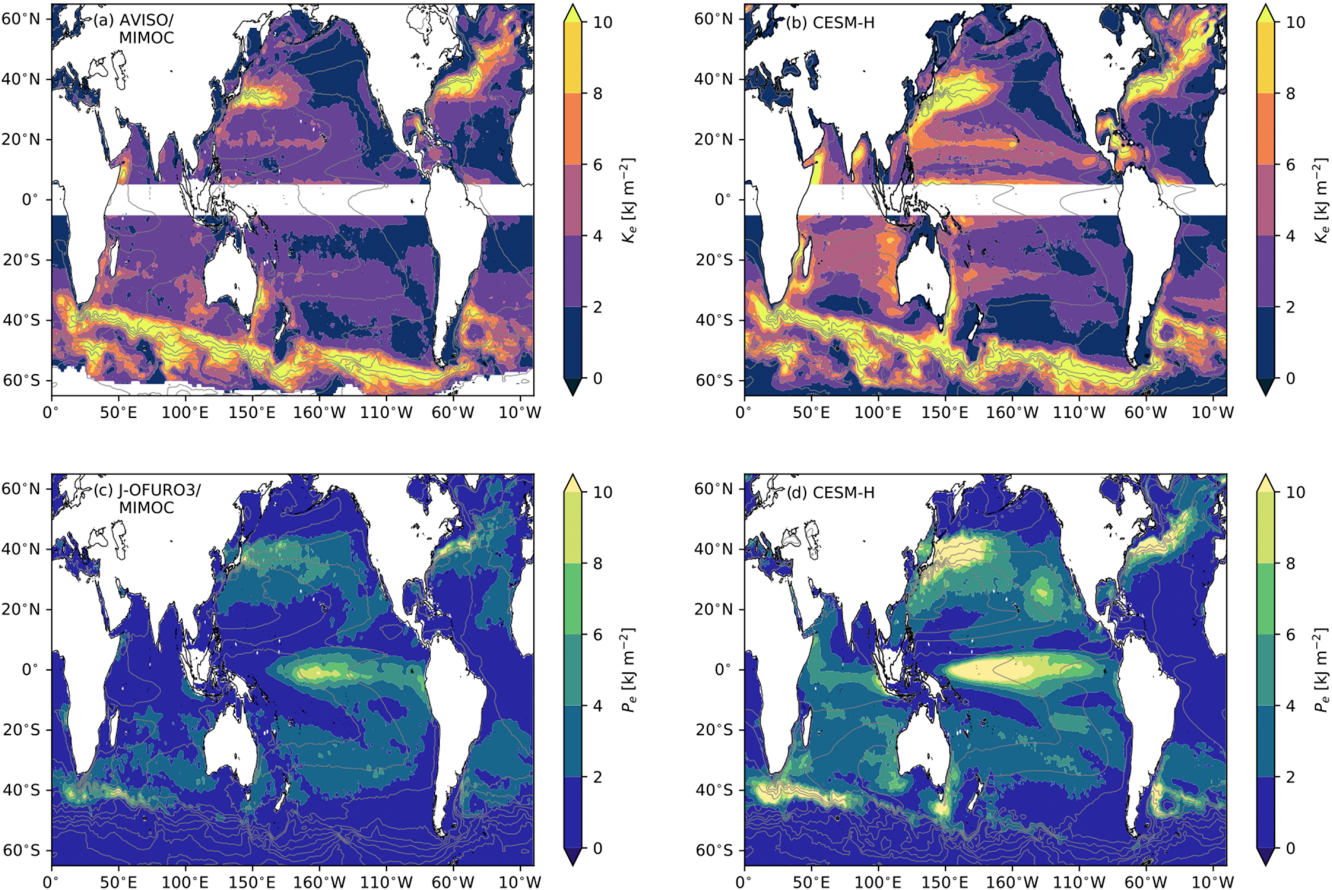
Eddy energetics per unit surface area from observational products and a mesoscale eddy‐resolving coupled climate model (CESM‐H) for the mean ML in units of kJ m^−2^ (kJ = 10^3^ J). (a) EKE per unit surface area from AVISO and MIMOC climatology. (b) Same as for (a) except for a model estimate using CESM‐H. (c) EPE per unit surface area from J‐OFURO3 and MIMOC climatology. (d) Same as for (c) except for a model estimate using CESM‐H. Color contours have contour intervals (ci) = 2 kJ m^−2^, and gray contours are mean SSH with ci = 25 cm.

We use the approximate estimates from (10) and (11) to quantify the typical magnitudes of the upper ocean energy, to compare observational and CESM‐H‐based estimates and to provide context with earlier studies (von Storch et al., 2012) in Figure 1. The EKE and EPE are highest in the ocean's western boundary currents, including the Gulf Stream and Kuroshio‐Oyashio Extension regions in the Northern Hemisphere, Agulhas Return Current, East Australian Current, and Brazil‐Malvinas Confluence in the Southern Hemisphere, and Antarctic Circumpolar Current (ACC). There are elevated regions in the North Atlantic along the Azores front, across the western and central Pacific near 20°N, the eastern tropical Pacific, and portions of the Indian Ocean such as the Somalia Current. The high mixed layer EKE and EPE in the North Atlantic is due to the North Atlantic Current and very deep mixed layer depths where mode water formation occurs away from boundary currents and regions of deep mixing. A notable energetic region is the high EKE west of the Hawaiian Islands (Figures 1a and 1b), likely due to wind‐driven eddies in the lee of the islands (Calil et al., 2008; Yoshida et al., 2010). The eastern tropical Pacific has moderate EKE and EPE maxima along the coast of mainland Mexico and Central America, attributed to the wintertime gap winds from the Gulf of Tehuantepec (southern mainland Mexico), Papagayo (Nicaragua), and Panama (Chang et al., 2012; Liang et al., 2012). The wind stress curl associated with these narrow jets leads to SST transient anomalies, which tend to vary with ENSO (Alexander et al., 2012).

**Figure 2:**
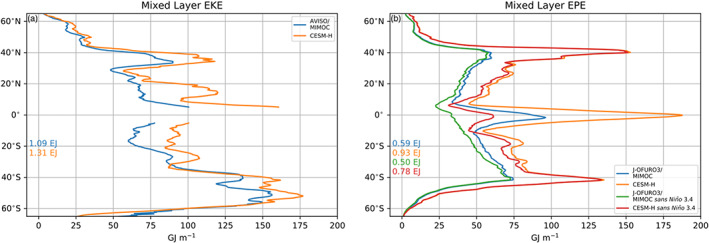
Zonal integral of eddy energetics in the ML in units of GJ m^−1^ (GJ = 10^9^ J). (a) Estimate of EKE from observations and CESM‐H with the global integral (±65° latitude) color coded in EJ (EJ = 10^18^ J). (b) Same as in (a) but for EPE and includes both observational and CESM‐H estimates with and without the Niño 3.4 index regression removed.

The zonally integrated mixed layer EKE from observations and CESM‐H are shown in Figure 2a. The zonally integrated EKE in observations and CESM‐H compare well, with CESM‐H having slightly higher values at most latitudes. The total globally integrated mixed layer EKE is 1.09 EJ in observations and 1.31 EJ in CESM‐H, which is about a third of the estimated total ocean EKE of 3.55 EJ (von Storch et al., 2012).

The zonally integrated mixed layer EPE has prominent peaks in the midlatitudes and the tropics (Figure 2b). The much higher EPE in CESM‐H is associated with elevated SST variance, which has been documented in Small et al. (2014). This bias is still not fully understood. The tropical mixed layer EPE is largely due to ENSO. When the Niño 3.4 index regression is removed the peak in the tropics is reduced significantly. The globally integrated mixed layer EPE is 0.59 and 0.93 EJ in observations and CESM‐H, respectively, compared with 0.5 and 0.78 EJ with the Niño 3.4 index regression is removed, which is about ∼15% reduction. Removing the Niño 3.4 index regression has almost no impact on the midlatitude distribution of EPE, indicating negligible impact of teleconnections on the energetics. The global value of EPE integrated through the full ocean depth obtained by von Storch et al. (2012) is 6.38 EJ, which is about 11 times our mixed layer estimate based on observations and 7 times larger than the estimate based on CESM‐H.

### 3.2 Three‐Way Decomposition of Local APE Generation

**Figure 3:**
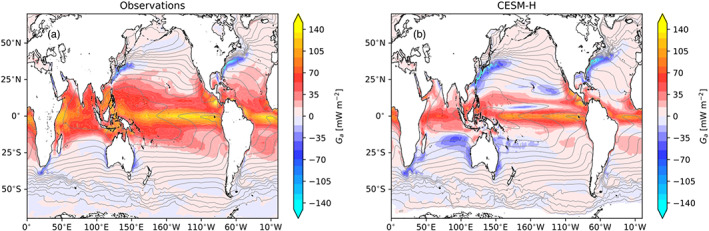
The local spatial pattern of APE generation (*G*_*a*_) from (a) observations (J‐OFURO3/MIMOC) and (b) CESM‐H with ci = 5 mW m^−2^. Black contours are mean SST (ci = 2°C) for J‐OFURO3 (a) and CESM‐H (b).

The local thermal component of APE generation before surface area integration, (3), is shown in Figure 3 from observations and CESM‐H. The high‐resolution coupled model has comparable spatial patterns to observations. The vast majority of generation occurs in the tropics with peak values in excess of 150 mW m^−2^ in the eastern tropical Pacific and tropical Atlantic in observations (Figure 3a) with smaller generation in CESM‐H (Figure 3b). The remainder of the ocean is a weak source of APE outside of the midlatitude western boundary currents, which are a significant spatially confined sink. CESM‐H exhibits a small sink in the central tropical Pacific and regions of the subtropics in the North Pacific east of the Hawaiian Islands, east of Australia, and within the Indian Ocean that do not appear in observations.

**Figure 4:**
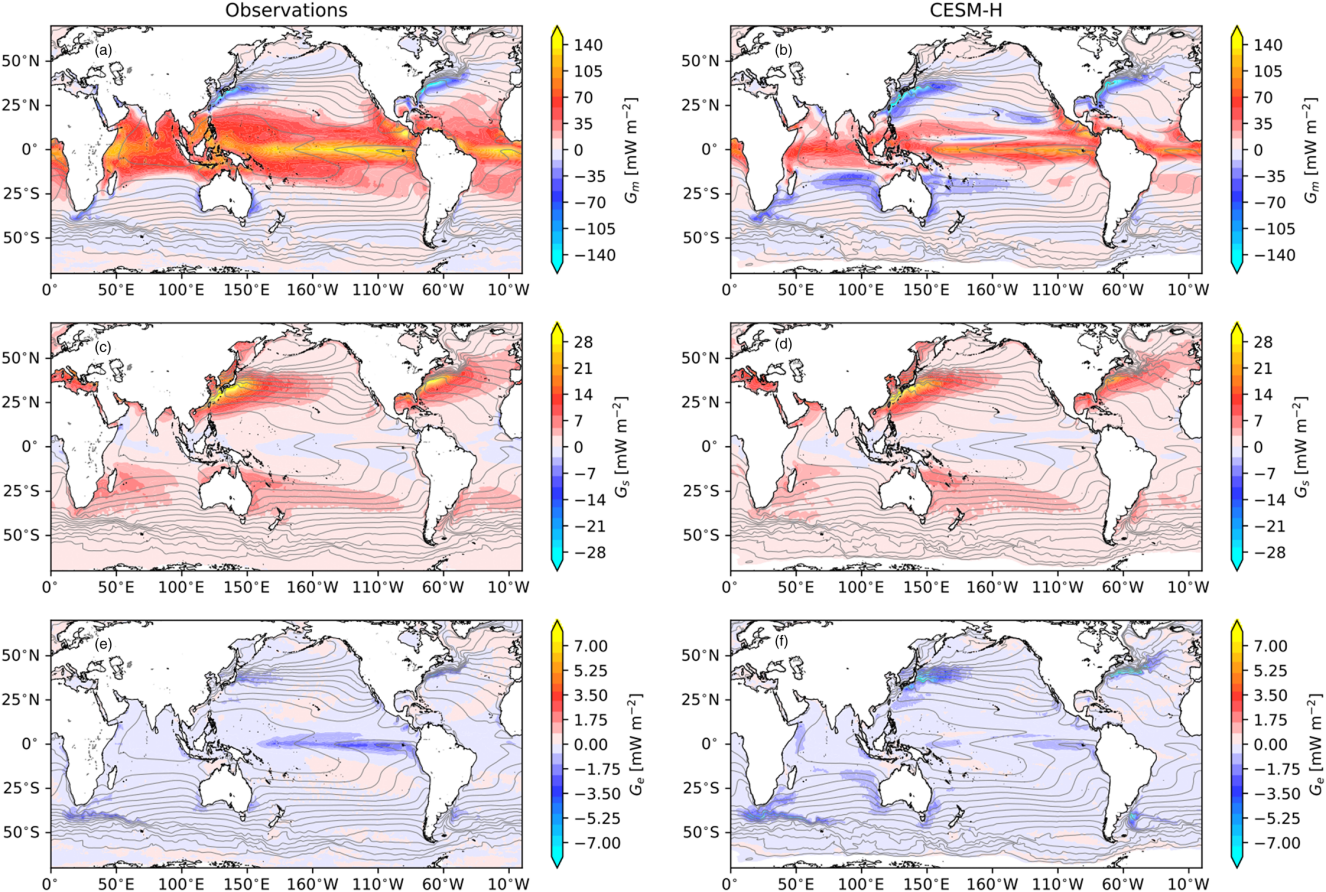
Three‐way decomposition of the local spatial pattern of APE generation in observations (J‐OFURO3/MIMOC) and CESM‐H. *G*_*m*_ in observations (a) and CESM‐H (b). *G*_*s*_ in observations (c) and CESM‐H (d). *G*_*e*_ in observations (e) and CESM‐H (f). Color contours with ci = 5, 1, and 0.25 mW m^−2^ for *G*_*m*_, *G*_*s*_, and *G*_*e*_, respectively. Black contours are mean SST (ci = 2°C) for J‐OFURO3 (left column) and CESM‐H (right column).

The three‐way decomposition of local APE generation, that is, Equations (5), (6), (7), is shown in Figure 4. The mean generation (*G*_*m*_) is shown in Figures 4a and 4b for observations and CESM‐H, respectively. The mean shares a striking spatial resemblance to the total generation in Figure 3, showing that a majority of the APE in the global ocean is generated through the mean state. The seasonal contribution (*G*_*s*_) is positive, which indicates that it is a source of APE (Figures 4c and 4d). The seasonal contribution is largest in the midlatitude and poleward regions of the Northern Hemisphere with values in excess of 30 mW m^−2^ in the Gulf Stream and Kuroshio Extension regions. The seasonal source is much smaller in the tropics and Southern Hemisphere. The transient eddy values are a sink of APE that is nearly 5 times smaller than the seasonal source (Figures 4c and 4d) and 10 times smaller than the mean (Figures 4a and 4b) and more tightly confined to the western boundary currents, ACC, and eastern tropical Pacific with peak values in excess of −8 mW m^−2^. The next section takes a closer look at the regional western boundary currents and Southern Ocean ACC with a comparison of the spatial patterns to the local mean SST and SSH gradients.

#### 3.2.1 Midlatitude Regional Hot Spots

##### 3.2.1.1 Northern Hemisphere Western Boundary Currents

**Figure 5:**
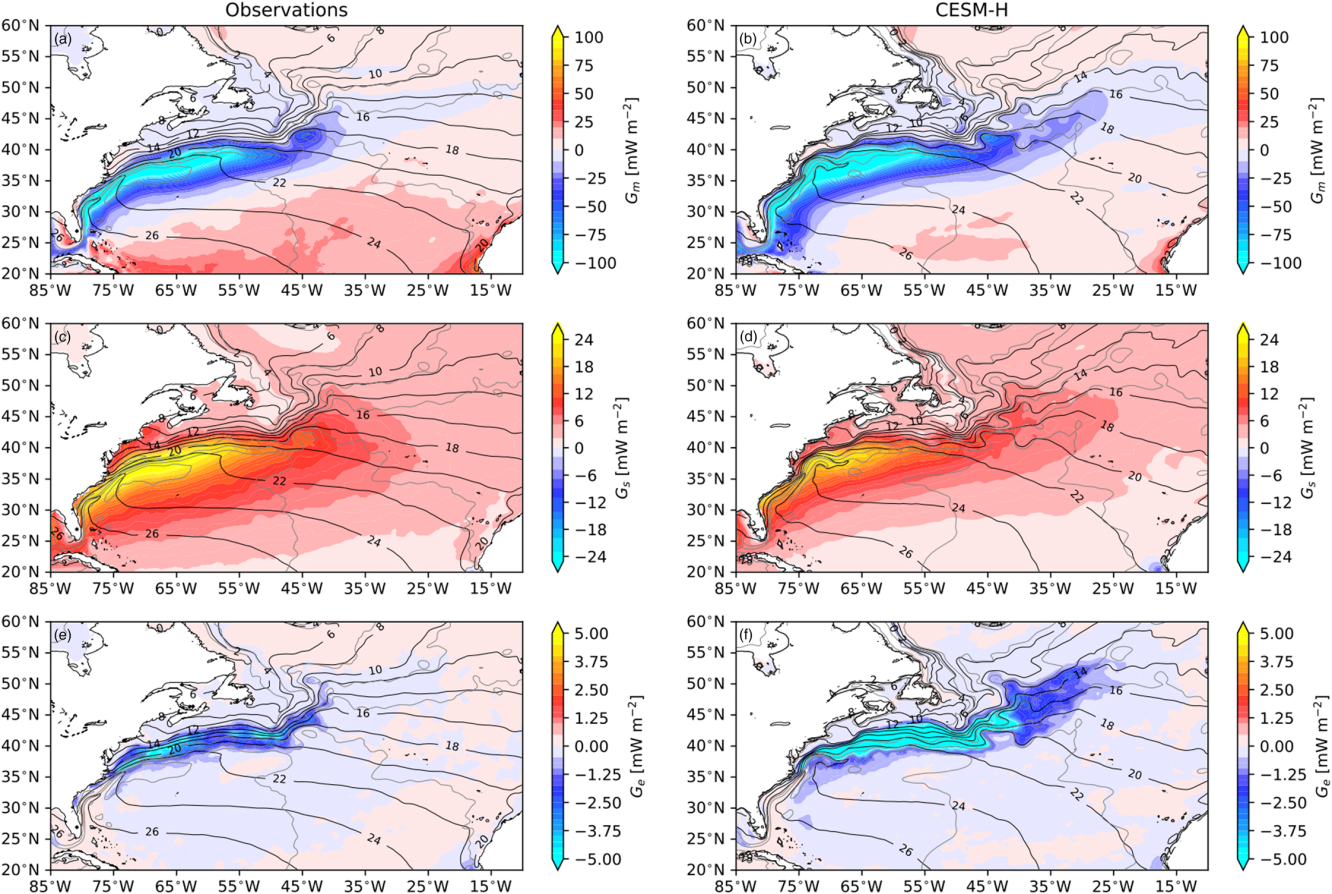
Gulf Stream region local APE generation from observations (J‐OFURO3/MIMOC left column) and CESM‐H (right column). *G*_*m*_ in observations (a) and CESM‐H (b). *G*_*s*_ in observations (c) and CESM‐H (d). *G*_*e*_ in observations (e) and CESM‐H (f). Color contours with ci = 5, 1, and 0.25 mW m^−2^ for *G*_*m*_, *G*_*s*_, and *G*_*e*_, respectively. Black contours are the mean SST (ci = 2°C), and gray contours are the mean SSH (ci = 25 cm) for J‐OFURO3 (left column) and CESM‐H (right column), respectively.

**Figure 6:**
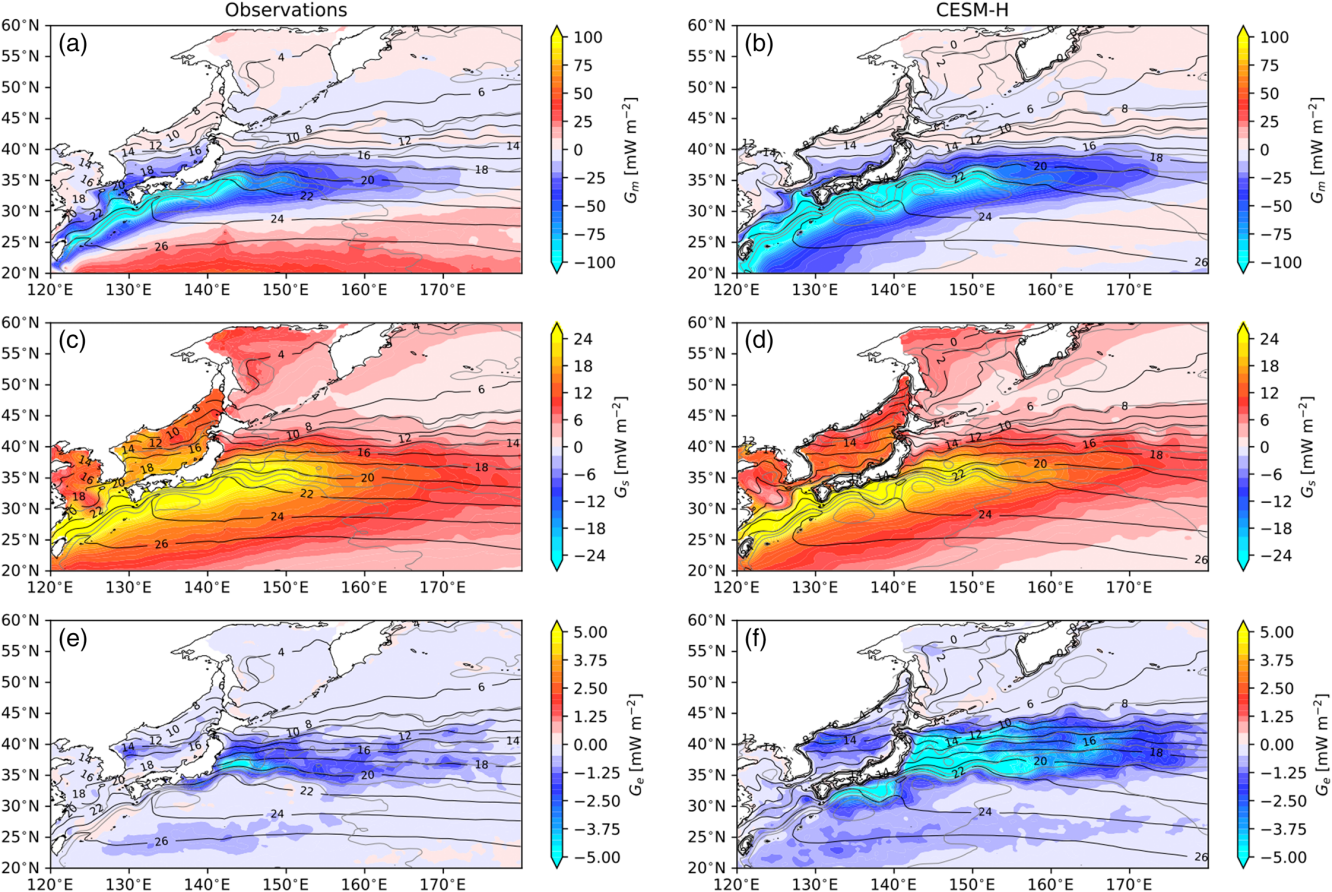
Same as in Figure 5 but for the KOE region.

**Figure 7:**
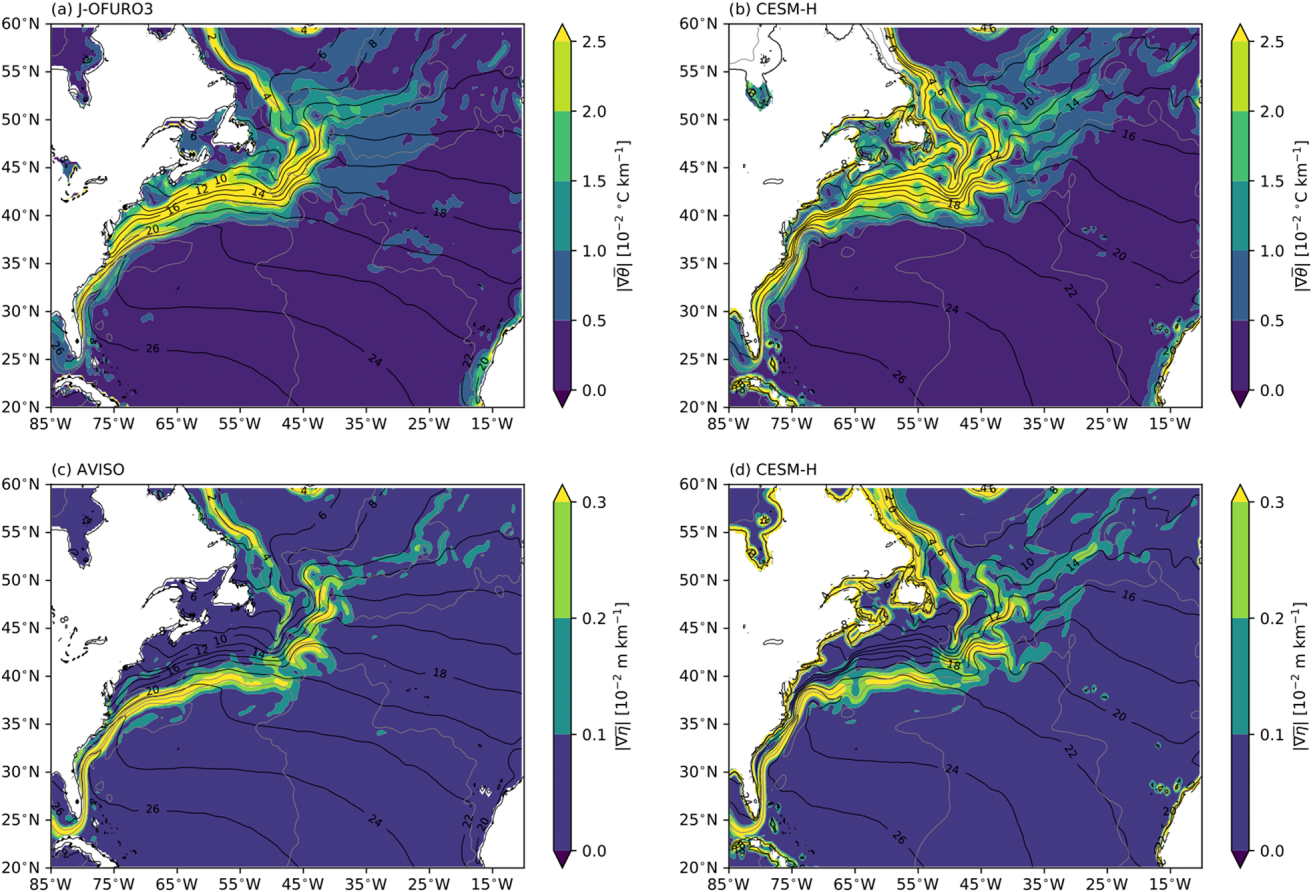
Mean gradient magnitudes from observations (left column) and CESM‐H (right column). SST gradient from J‐OFURO3 (a) and CESM‐H (b). SSH gradient from AVISO (c) and CESM‐H (d). Color contours are ci = 0.5°C per 100 km for SST gradients and ci = 0.1 cm per 100 km. Black contours are the mean SST (ci = 2°C), and gray contours are the mean SSH (ci = 25 cm) for observations (left column) and CESM‐H (right column), respectively.

The local *G*_*m*_, *G*_*s*_, and *G*_*e*_ are shown for the Gulf Stream (Figure 5) and Kuroshio Extension (Figure 6) regions. A closer look at the Gulf Stream and Kuroshio Extension shows that *G*_*m*_ and *G*_*s*_ are closely tied to the warmest SST core and mean flow on the southern portion of each current system. The *G*_*e*_ sink is preferentially located on the northern side of each current system. The mean SST and SSH gradients for the Gulf Stream are shown in Figure 7. It can be seen that the *G*_*m*_ and *G*_*s*_ are associated with the warm core of the Gulf Stream within and southwest of the SSH gradients, while *G*_*e*_ is associated more closely with SST gradients to the north. The Kuroshio Extension shows the same spatial relationships between APE generation and the mean SST and SSH gradients.

##### 3.2.1.2 Southern Ocean

**Figure 8:**
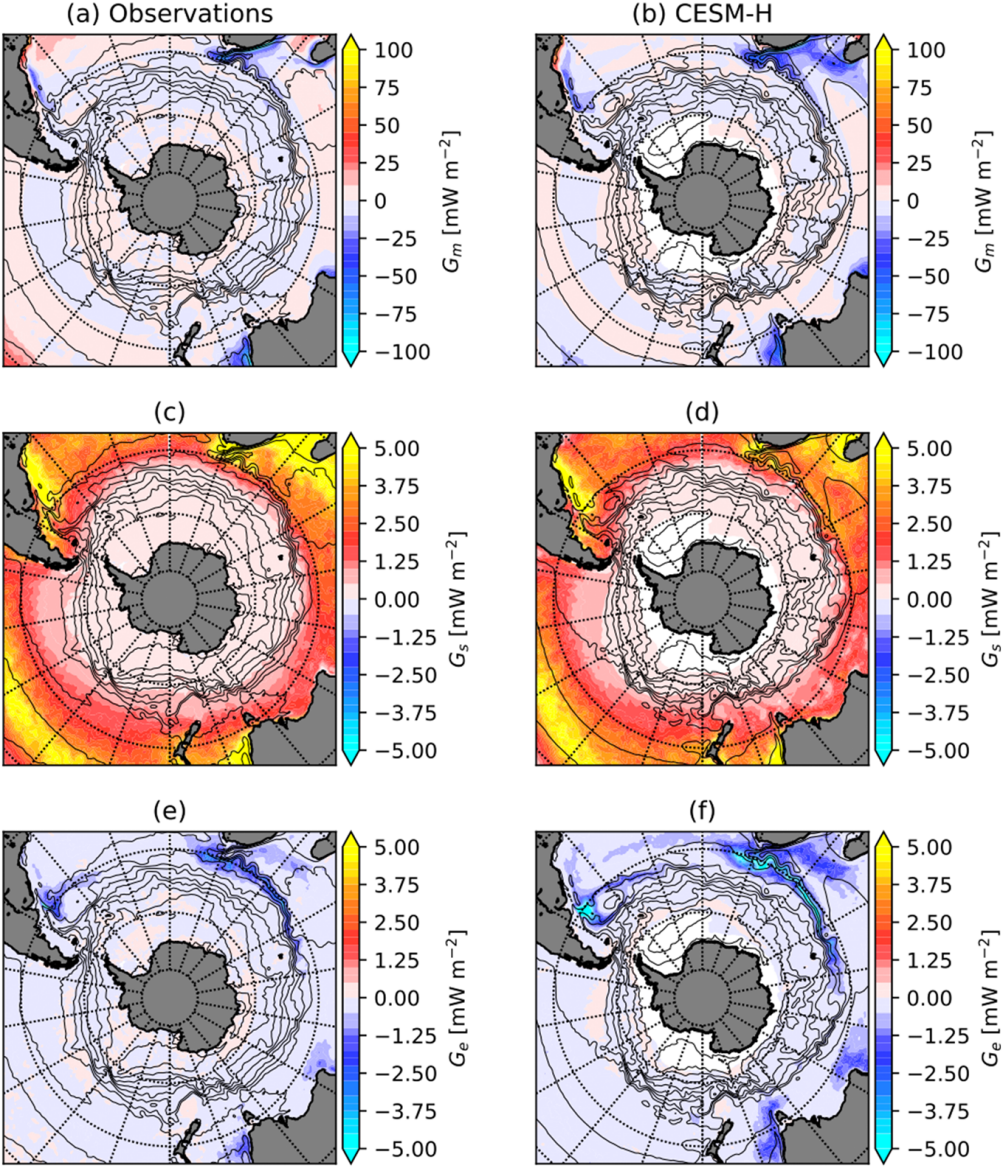
Local thermal available potential energy generation in the Southern Ocean south of 35°S from observations (J‐OFURO3/MIMOC) (left column) and CESM‐H (right column). MPE generation (*G*_*m*_) in observations (a) and CESM‐H (b). SPE generation (*G*_*s*_) in observations (c) and CESM‐H (d). *G*_*e*_ in observations (e) and CESM‐H (f). Color contours with ci = 5 mW m^−2^ for *G*_*m*_ and 0.25 mW m^−2^ for *G*_*s*_ and *G*_*e*_. Note that *G*_*s*_ and *G*_*e*_ are on the same colormap scale. Black contours are the mean SSH (ci = 25 cm) for observations (left column) and CESM‐H (right column), respectively.

*G*_*m*_, *G*_*s*_, and *G*_*e*_ are shown for the Southern Ocean ACC in Figure 8. As in the Northern Hemisphere western boundary currents *G*_*m*_ is mostly negative, indicating a sink of APE in the Southern Hemisphere, that is, Agulhas Return Current and Brazil‐Malvinas Confluence, with the model agreeing well in magnitude and spatial location (Figures 8a and 8b). *G*_*m*_ is very weak outside of the western boundary currents along the ACC path.

**Figure 9:**
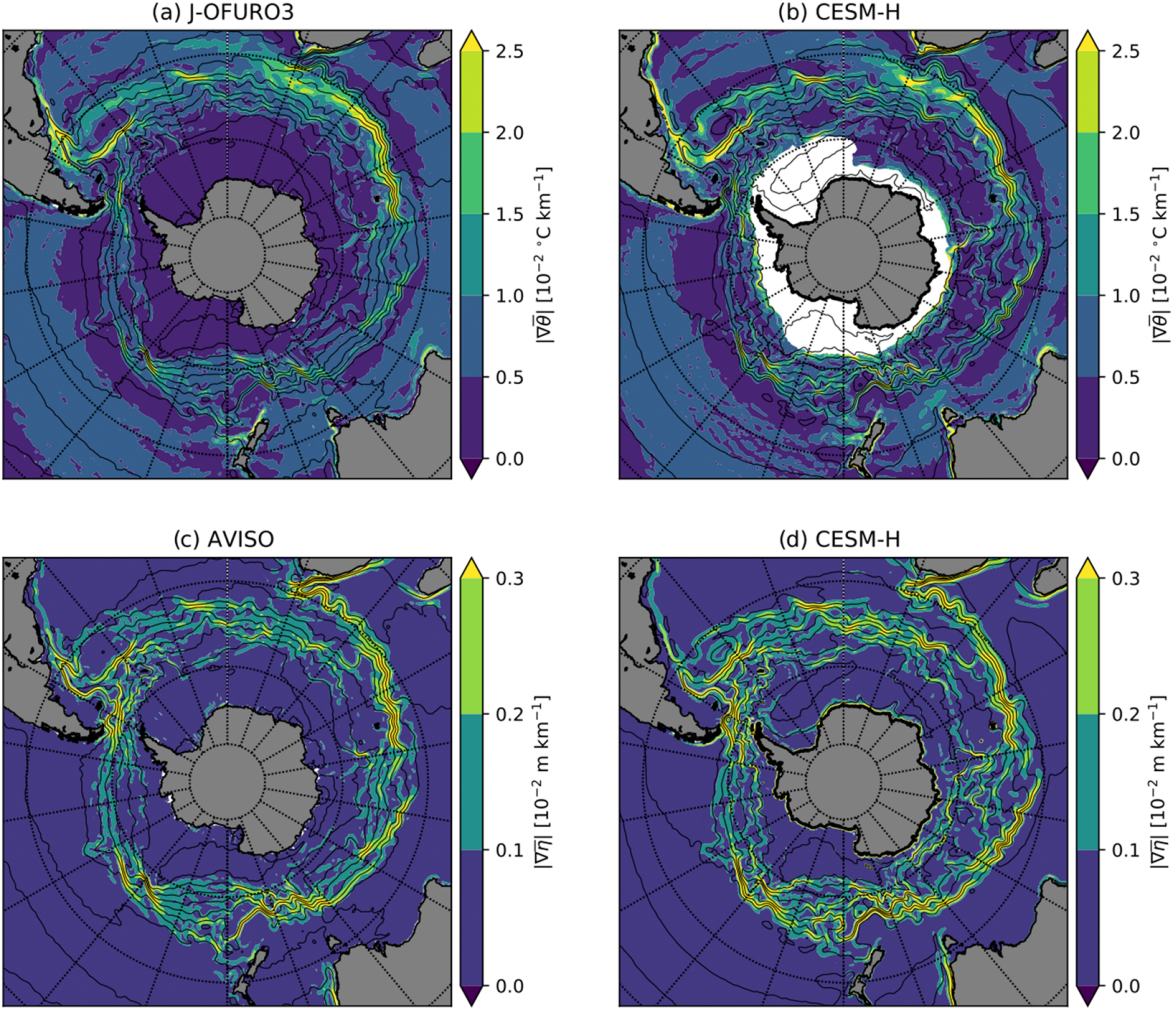
Southern Ocean mean gradient magnitudes from observations (left column) and CESM‐H (right column). SST gradient from J‐OFURO3 (a) and CESM‐H (b). SSH gradient from AVISO (c) and CESM‐H (d). Color contours are ci = 0.5°C per 100 km for SST gradients and ci = 0.1 cm per 100 km. Black contours are the mean SSH (ci = 25 cm) for observations (left column) and CESM‐H (right column), respectively.

*G*_*s*_ is much smaller in the Southern Hemisphere compared with the Northern Hemisphere (Figures 4e and 4f), but it is the same magnitude as *G*_*e*_ (Figures 8c and 8d). The *G*_*s*_ source is strongest near the continents and within midlatitude subtropical gyres north of the ACC. Within the ACC there are regions of near‐zero *G*_*s*_. *G*_*e*_ (Figures 8e and 8f) is a sink and largest in the Southern Hemisphere western boundary currents: Agulhas Return Current, Brazil‐Malvinas Confluence, and East Australian Current systems. An elevated sink is observed along the southwestern coast of Australia, likely associated with the Leeuwin Current variability. Similar to the Northern Hemisphere, there is a notable offset in spatial location with *G*_*m*_ and *G*_*s*_ aligned with mean SSH gradients and *G*_*e*_ with SST gradients (Figure 9).

### 3.3 Globally Integrated Available Potential Energy Generation

**Figure 10:**
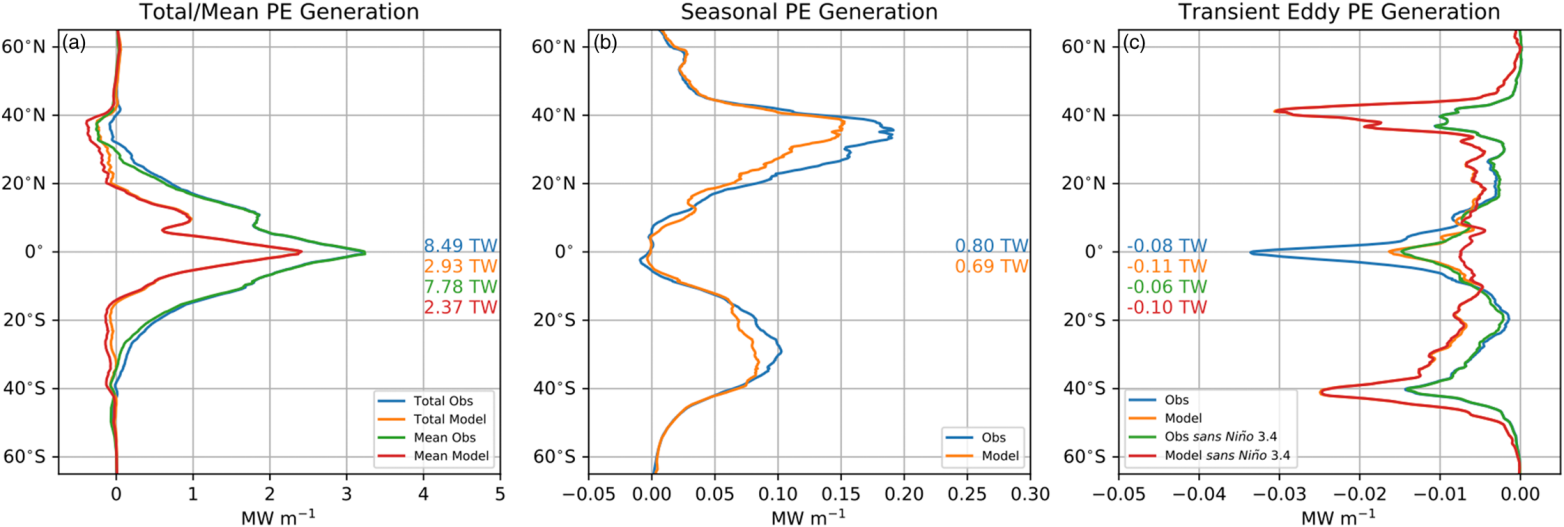
Zonally integrated available potential energy rate of generation from observations and CESM‐H. (a) *G*_*a*_, (b) *G*_*s*_, and (c) *G*_*e*_. Units are in MW m^−1^ (MW ≡ 10^6^ W). Global available potential energy generation rates are reported in TW (TW ≡ 10^12^ W) for each term in (a)–(c) color coded from the legend.

The zonally integrated rate of APE generation is shown in Figure 10. *G*_*a*_ and *G*_*m*_ rates are large and positive and mostly confined to the ±20° latitude where they are indistinguishable. The peak in generation occurs at the equator at >3 MW m^−1^ (MW = 10^6^ W) in observations and >2 MW m^−1^ in CESM‐H and decays toward the poles (Figure 10a). The surface area integrated rate of total APE generation is positive and 8.49 TW (TW = 10^12^W) from observations and 2.93 TW from CESM‐H. *G*_*m*_ is very close to the total in the tropics (±20° latitude) but is less than the total and negative in the midlatitudes. The surface area integrated rate of mean generation is 7.78 and 2.37 TW from observations and CESM‐H, respectively.

The difference in the total and mean APE rate of generation is accounted for by the seasonal and transient eddy contributions (Figures 10b and 10c). The seasonal rate of generation is positive and largest in the midlatitude Northern Hemisphere with peaks near 35°N at 0.19 and 0.15 MW m^−1^ in observations and CESM‐H, respectively. The Southern Hemisphere peaks near 30°S with values of 0.1 and 0.08 MW m^−1^ in observations and CESM‐H, respectively. The *G*_*s*_ rate is larger in the Northern Hemisphere than in the Southern Hemisphere and is near zero in the tropics. The surface area integrated rate of seasonal generation is positive and 0.8 TW from observations and 0.69 TW from CESM‐H.

*G*_*e*_ is a sink at all latitudes and has three latitudinal peaks at the equator and midlatitudes (Figure 10c). The model estimate is larger in midlatitudes but is smaller in the tropics. More than half of the tropical EPE destruction is due to ENSO. After removing a regression of the Niño 3.4 index the tropical sink reduced at the equator by 55% in observations and 56% in CESM‐H. There is no detectable impact of ENSO feedbacks in midlatitudes.

In contrast to the tropics, the midlatitude *G*_*e*_ rate is larger in CESM‐H than in observations in both hemispheres. In observations the sink is larger in the Southern Hemisphere than in the Northern Hemisphere midlatitudes but is the opposite in CESM‐H with a larger sink occurring in the midlatitude Northern Hemisphere than in the Southern Hemisphere. The peak is near 40° latitude in both hemispheres. This shows a preference for the destruction of EPE to occur poleward of *G*_*s*_ generation and alignment with the mean SST gradients versus SSH gradients.

The surface area integrated *G*_*e*_ rate is negative with values of −0.08 and −0.11 TW in observations and CESM‐H, respectively, showing that air‐sea interaction in the model is destroying more EPE than in observations. Latitudinally, the tropics (±20° latitude) account for 55.27% and 31.18% of the total globally integrated EPE rate of destruction in observations and CESM‐H, respectively. The Northern Hemisphere north of 20°N accounts for 18% and 32%, and the Southern Hemisphere south of 20°S accounts for 26.4% and 36.8% in observations and CESM‐H, respectively. This reiterates that the model has a weaker EPE destruction in the tropics and a stronger destruction in the midlatitudes. When the Niño 3.4 regression is removed the EPE destruction is −0.06 and −0.1 TW in observations and CESM‐H. Globally, EPE destruction associated with ENSO is approximately −0.02 TW (25%) in observations and only −0.01 TW (9%) in CESM‐H.

**Figure 11:**
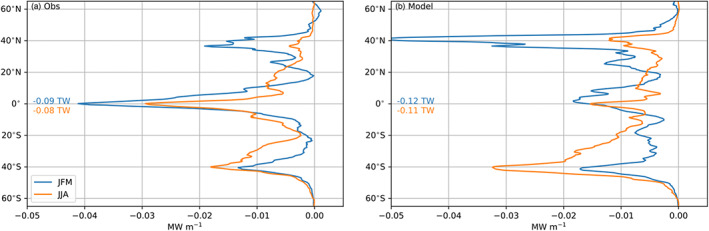
Zonally integrated EPE sink from observations (a) and CESM‐H (b) partitioned by season. Blue is the JFM (January, February, and March) average and orange JJA (June, July, and August) average. Units are in MW m^−1^, and global available potential energy generation rates are reported in TW for each term and color coded from the legend as in Figure 10. ENSO has not been removed for these estimates.

The three‐way decomposition of the APE generation does not mean that the transient eddies do not have a seasonal cycle. Figure 8 in Bishop et al. (2017) shows that the covariance between SST and turbulent (i.e., latent and sensible) heat fluxes is strongest in winter. The dissipation of SST variance in CESM‐H was also found to be strongest during winter in the Kuroshio Extension region (Yang et al., 2019). This implies that SST, turbulent heat flux anomalies, or both are enhanced during the winter months, which is consistent in the western boundary currents where winds and net heat fluxes are largest during winter. Figure 11 shows the transient eddy sink of APE partitioned between JFM (January, February, and March) and JJA (June, July, and August) averages for observations (Figure 11a) and CESM‐H (Figure 11b). The observations show three latitudinal peaks for JFM (Figure 10c). The midlatitude Northern Hemisphere sink is notably stronger during the Northern Hemisphere winter months. The equatorial sink is also larger during JFM, which is when ENSO peaks in amplitude. The difference between the Southern Hemisphere winter (JJA) and summer (JFM) is less pronounced but is a larger sink during winter. The CESM‐H (Figure 11b) has a much stronger midlatitude seasonal change in *G*_*e*_ than in observations (Figure 11a) with a larger sink during winter for each respective hemisphere. However, the tropical sink is less pronounced and does not have as clear of a seasonal change as in observations.

### 3.4 Scale Dependence of EPE Generation

**Figure 12:**
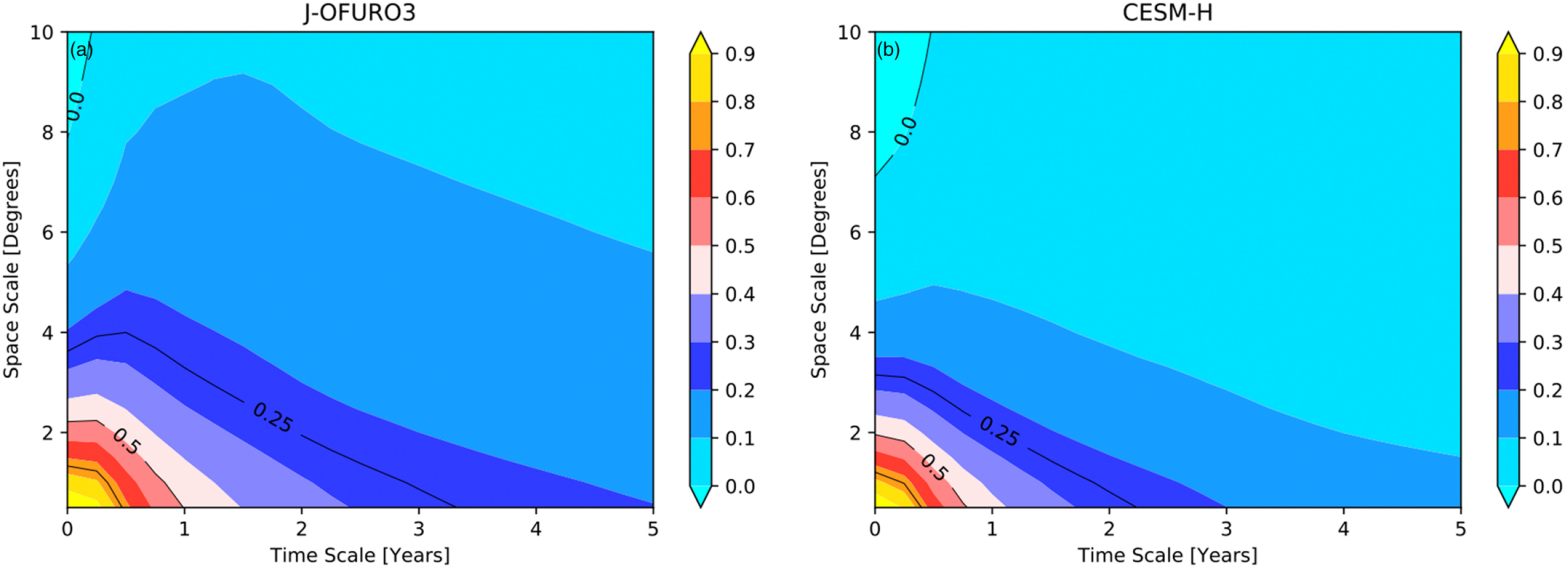
Space‐scale versus timescale dependence of the global *G*_*e*_ rate *sans* Niño represented as a percentage of the unfiltered rate. (a) J‐OFURO3 and (b) CESM‐H. Color contours have ci = 0.1, and black contours have ci = 0.25.

In Bishop et al. (2017) it was shown that the correlation between SST and air‐sea turbulent heat fluxes (sensible plus latent) has a strong space‐scale and timescale dependence. In order to assess the scale dependence of the global EPE sink by air‐sea interaction we adopt a similar method for determining the scale dependence by smoothing the SST and net heat flux anomaly fields in space and time as described in section 2.5 before estimating (7). The ratio of the filtered to unfiltered global surface area integrated *G*_*e*_ rate (Figure 12) is a nonlinear function of space scale versus timescale. Figure 12a shows that the observations maintain 50% of the EPE destruction rate for annual timescales and spatial scales of ∼2.25°. At timescales up to 5 years 20% of the EPE destruction rate is still maintained for spatial scales less than 2°. As spatial scales are smoothed from 4° to 8°, the *G*_*e*_ filtered to unfiltered ratio is reduced from 20% to near zero. CESM‐H exhibits similar behavior but decays in space and time more quickly than observations (Figure 12b). At annual timescales only ∼40% of the EPE destruction rate is maintained and 50% at 2° spatial scales. At 4° of spatial smoothing there is only 10–20% of EPE destruction remaining. The zero crossing occurs at ∼7° in CESM‐H versus 8° in observations.

## 4 Discussion

**Figure 13:**
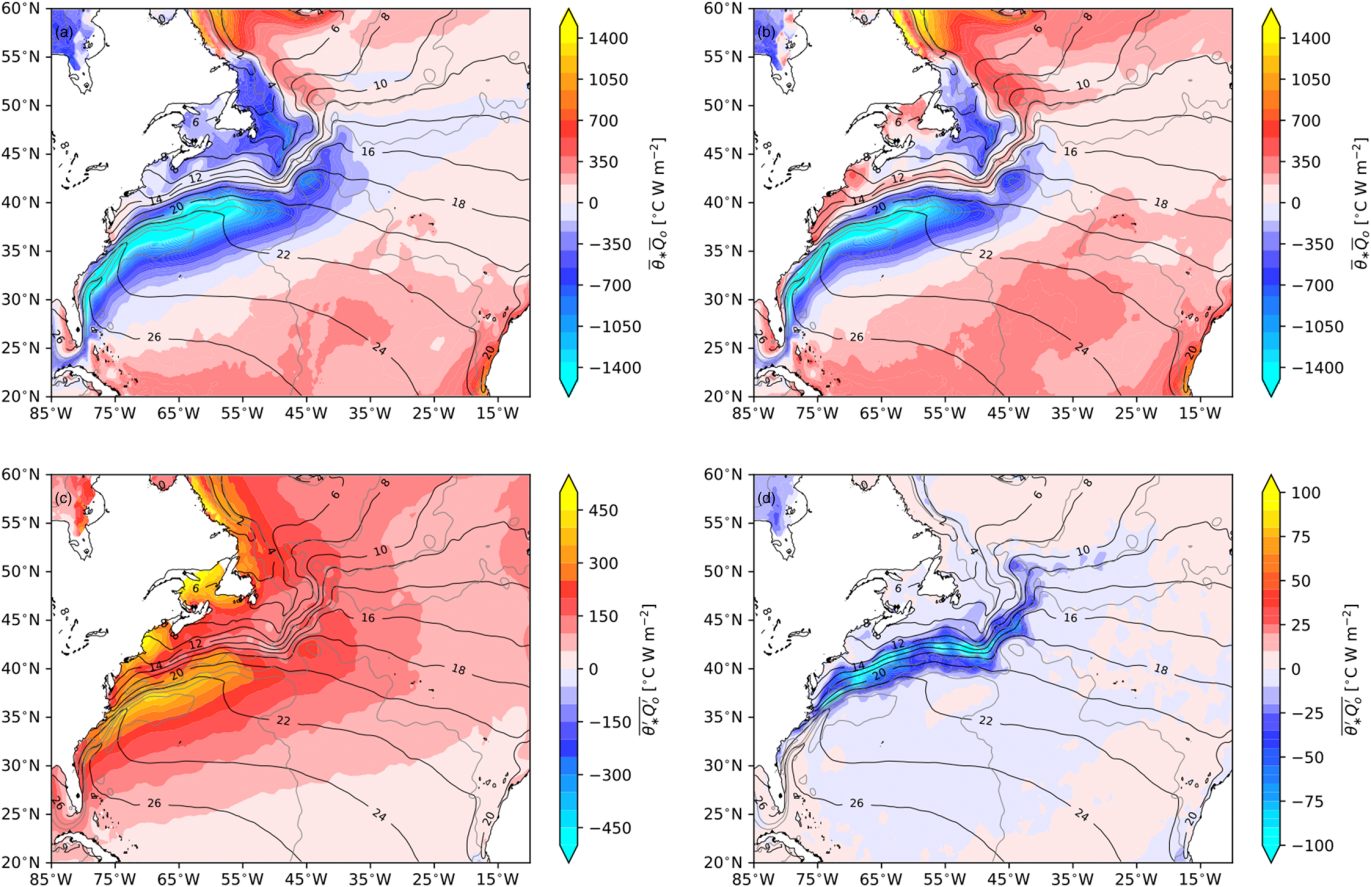
$\bar{\theta_{*}Q_{o}}$ Reynolds decomposition in the Gulf Stream from J‐OFURO3. (a) Mean term with no seasonal climatology, (b) mean term with seasonal climatology, (c) transient eddy term with seasonal climatology, and (d) transient eddy term with no seasonal climatology. Black contours are the mean SST (ci = 2°C), and gray contours are the mean AVISO SSH (ci = 25 cm).

In von Storch et al. (2012) eddy terms in the energy budget were derived using anomalies defined as deviations from the time mean and included all timescales. A major distinction made in this study is that the eddy APE generation term is derived with anomalies defined as deviations from a mean state, which includes the mean monthly climatology rather than simply the time mean. When the mean monthly climatology is removed, this effectively removes the mean seasonal cycle, which is a forced pattern not associated with ocean dynamics and internal variability. To illustrate the difference between using a mean state as the time mean or the mean monthly climatology, Figure 13 shows the covariance of SST and net heat flux in the Gulf Stream region from J‐OFURO3. In Figures 13a and 13c the Reynolds decomposition is partitioned between the mean and eddy terms with the mean state being the time mean, which will be referred to as the “traditional” decomposition. In Figures 13b and 13d the mean state is the mean monthly climatology as in this paper, which includes the mean seasonal cycle. The transient eddy term is very different depending on the decomposition of mean and anomaly. The nature of transient eddies to remove APE through air‐sea interaction is masked in the traditional decomposition, which is misleadingly positive, attributing mesoscale air‐sea interaction as a source of APE (von Storch et al., 2012). In this work we can see that the ocean dynamics associated with internal variability acts to remove APE through air‐sea interaction (Figure 13d). The timescale and space‐scale dependence of *G*_*e*_ (Figure 12) implies that at spatial scales encompassing the mesoscale (<2°) the EPE sink extends to interannual and longer timescales. Places such as the Kuroshio Extension, which exhibits bimodality in its meandering states on decadal timescales (Qiu & Chen, 2005), are good examples of potential causes of the longer timescale modulation.

As was mentioned earlier and pointed out in previous studies, that is, (Bishop et al., 2017), removing the mean seasonal cycle does not imply that the transient eddies do not have a seasonal signal. The main impacts of winds and net air‐sea heat loss over the western boundary currents are felt mainly during the winter months. The presence of mesoscale SST anomalies during winter, derived from ocean internal variability, has known effects on wind stress (Chelton et al., 2004). To this effect it is not surprising that Figure 11 shows the wintertime (JFM) enhancement of the EPE sink in the midlatitudes in both hemispheres over summer months (JAS), when lighter winds prevail, and net heat is gained by the ocean. The importance of the three‐way decomposition (4) in removing the mean seasonal cycle is important globally at the surface but is a notably weaker contribution in the Southern Ocean. By comparing the magnitude of the transient eddy to seasonal APE generation there are only small regions in the Northern Hemisphere where the transient eddy signal is upwards of 25% of the seasonal signal within the Gulf Stream SST front. However, the transient signal is equal to or stronger than the seasonal signal within portions of the Agulhas Return Current and Brazil‐Malvinas Confluence but smaller along other portions of the ACC.

One aspect of this study that is missing is the contribution of transient sea surface salinity and freshwater fluxes to mesoscale EPE generation or destruction. From von Storch et al. (2012) it was found that the freshwater flux is much less efficient in changing density than heat flux. The contribution from freshwater fluxes reaches a local maximum generation in the tropics of 20 mW m^−2^ from excess precipitation over evaporation. In contrast to our study the seasonal cycle of transient eddy salt transport in the North Atlantic is small everywhere except in the tropics (Treguier et al., 2012). The availability of sea surface salinity and freshwater flux observations is lacking compared with SST and net heat flux, but future work will examine their contribution to APE generation.

An interesting result of this paper is that the transient eddy removal of APE through air‐sea interaction is spatially offset from the energy removed by the mean flow in the western boundary currents. An examination of the SST and SSH mean gradients shows that the transient eddy removal of APE is aligned with SST gradients and the removal of mean PE with SSH gradients within and equatorward of the respective western boundary current main jets and aligned with the climatological net heat flux loss. The difference between SSH and SST gradients in the Kuroshio Extension region was explored in Jing et al. (2019) and Qiu et al. (2017). The close correspondence of the air‐sea EPE sink with mean SST gradients points to frontal instabilities and mixing length arguments (Stammer, 1998) that contribute to anomalous air‐sea interaction in these regions. It also shows a preference for the sink of EPE to occur on the poleward side of the main western boundary currents rather than the southern side and begs the question as to which types of eddies are most important in removing EPE? This has implications for climate modeling since there is no explicit EPE in standard coarse‐resolution climate models but is parameterized with no current ability to mimic the eddy effects on air‐sea feedbacks. The observational data sets are inadequate to perform a full Lorenz energy cycle as in von Storch et al. (2012), but ongoing work shows that the mesoscale air‐sea EPE sink is an O(1) term in the upper ocean EPE budget compared with estimates of baroclinic conversion rates (Guo & Bishop, personal communication 2020). The missing parameterized air‐sea interaction in standard climate models may have unforeseen effects on the ocean circulation and overlying atmospheric storm tracks, which deserve further study.

Some of the missing processes in standard climate models occur in the western boundary currents, which exhibit a wide range of variability from waves and meanders to cutoff rings on the northern side (warm‐core rings) and southern side (cold‐core rings) of the main currents. This work suggests that there may be a difference between cold‐core rings and warm‐core rings with cold core having very little impact on air‐sea interaction as a sink of EPE in the subtropical gyres. Figure 12 in Sasaki and Minobe (2015) shows that SST anomalies associated with warm‐core rings are concentrated on the northern side of the Kuroshio Extension off the northern coast of Japan and shares a strikingly similar spatial pattern of mesoscale air‐sea removal of EPE shown in Figure 6e. Perhaps over warm‐core rings the lower atmosphere boundary layer is unstable versus cold‐core rings, which may aid in enhancing turbulent heat fluxes and wind anomalies (Chelton et al., 2004). Ma et al. (2016) further show a difference in the composite of turbulent heat fluxes over warm‐core versus cold‐core rings. Future work will explore what percentage of the mesoscale air‐sea sink of APE is due to waves and meanders versus cyclonic and anticyclonic vortices in the western boundary currents, following the techniques described in Gaube and McGillicuddy (2017), which made distinctions and found an asymmetric response of chlorophyll in the Gulf Stream to these different types of eddies.

Both cold‐core and warm‐core rings have been observed to decay over time in association with a decrease in APE over their lifetimes (Cheney & Richardson, 1976; Okada & Sugimori, 1986). It is interesting to note from Okada and Sugimori (1986) “For variability over short‐term periods, APE and kinetic energy are both decreased by air‐sea interaction because of a passing cyclone over the warm eddy area during the observation period.” Warm‐ and cold‐core eddies are currently absent from standard coupled climate models that are run at coarse resolution, and there are no parameterizations that take into account these features and their anomalous air‐sea interaction. If warm‐core rings are one of the main features that drive this anomalous air‐sea interaction that is a sink of EPE, the design of future parameterizations of these features must incorporate their life cycle. One potential pathway forward in parameterizing mesoscale air‐sea feedbacks may be through stochastic parameterization (Grooms et al., 2015) and coupling backscatter with the GM parameterization (Bachman, 2019).

## 5 Conclusions

In this work it is demonstrated that mesoscale air‐sea interaction is a global sink of APE with a magnitude of 0.1 TW in both observations and a high‐resolution version of the Community Earth System Model. The destruction of EPE through air‐sea interaction is confined to the tropics and midlatitude western boundary currents and portions of the Southern Ocean ACC. Locally, values of the EPE sink in the western boundary currents are O(10 mW m^−2^), which are consistent with values in the Kuroshio Extension (Ma et al., 2016). CESM‐H exhibits similar spatial structure compared with estimates from observations (J‐OFURO3) but is a larger sink in the midlatitude western boundary currents than in the tropics. After attempting to remove the instantaneous impacts of the ENSO, by regressing the Niño 3.4 index, it is shown that the impacts of ENSO are confined to the tropics between ±20° latitude with negligible teleconnections to the midlatitude western boundary currents.

An important distinction made in this work, compared to global estimates made in von Storch et al. (2012), is that the mean seasonal cycle is not included in the transient eddy term, which is a forced pattern and not associated with internal variability. The EPE generation term in von Storch et al. (2012) is primarily attributed to the seasonal cycle and deceptively appears as a source of EPE, particularly in the Northern Hemisphere midlatitudes and higher latitudes (Figure 10b). Scale dependence of the globally integrated sink of EPE shows that about 50% of the air‐sea impacts are at spatial scales less than 2° and annual timescales. The remaining energy sink within spatial mesoscale band (less than 2°) is in longer timescales, pointing to the modulation of the mesoscale on interannual and longer timescales.

When the APE is partitioned into mean, seasonal, and transient eddy contributions (4) there is an overwhelming input of energy in the tropics in the form of mean generation (Figure 10a) that is 10 times larger than EPE destruction globally (Figure 10c). For western boundary currents the mean, seasonal, and transient eddy contributions are spatially offset. The mean APE generation (*G*_*m*_) is a net sink of APE in the midlatitudes and is located where the current is intense (large gradients of SSH): It corresponds to the strong loss of enthalpy from the ocean over the warm currents. The seasonal APE generation (*G*_*s*_) is instead a net source of APE, still located within the intense current, and it arises from the fact that during wintertime, seasonally cold SSTs are associated with large heat losses due to enhanced midlatitude winds during winter. Finally, the EPE generation (*G*_*e*_) is a sink of APE, located at the SST front, and is linked to the fact that warm eddies are linked to intense heat fluxes out of the ocean, possibly because the warm SSTs destabilize the atmospheric boundary layer resulting in more intense surface winds.

There are potential implications for what types of eddies (i.e., cold‐ or warm‐core eddies) are most important in removing APE from the large‐scale circulation and what features of the mean circulation can possibly be used to parameterize anomalous air‐sea interaction in coarse‐resolution climate models. Great advances have been made in coupled climate models that can simulate the large‐scale turbulence of the ocean and their impacts on air‐sea interaction. However, it is uncertain how long it will take until these features are routinely incorporated into coupled climate simulations, and thus, they will need to continue to be parameterized in many applications.

## Data Availability

All data sets are publicly available. J‐OFURO3 data are archived online (https://j‐ofuro.scc.u‐tokai.ac.jp/en/). MIMOC data are archived online (https://www.pmel.noaa.gov/mimoc/). Sea surface height data were gathered online (https://www.aviso.altimetry.fr/en/data/products/sea‐surface‐height‐products/global/gridded‐sea‐level‐anomalies‐mean‐and‐climatology.html). CESM‐H data are archived on the Earth System Grid (https://www.earthsystemgrid.org/dataset/ucar.cgd.asd.hybrid_v5_rel04_BC5_ne120_t12_pop62.html).

## Appendix A Derivation of Three‐Way Decomposition

### A.1

The three‐way decomposition of the APE generation into mean, seasonal, and transient eddy contributions using discrete monthly archived data is achieved by first sorting the SST anomaly from a reference state (*θ*_∗_) and net air‐sea heat flux (*Q*_*o*_) into months by year:

(A1) $$\theta_{*}(x,y,t)\to \theta_{*}^{n}(x,y,\tau ),$$

(A2) $$Q_{o}(x,y,t)\to Q_{o}^{n}(x,y,\tau ).$$

$\theta_{*}^{n}(x,y,\tau )$ and $Q_{o}^{n}(x,y,\tau )$ are the sorted SST and net heat flux, respectively, *n* is year from 1, 2, …, *N* (*N* is the total number of years, *N* = 21 for observations and *N* = 35 for CESM‐H), and *τ* is month of the year from 1, 2, …, *M* (*M* ≡ 12). For this study the data were sorted using Python xarray module and its built‐in groupby commands. After sorting the SST and net heat flux they can both be written as

(A3) $$\theta_{*}^{n}(x,y,\tau )=\theta_{*}^{clim}(x,y,\tau )+\theta_{*}^{n\prime }(x,y,\tau ),$$

(A4) $$Q_{o}^{n}(x,y,\tau )=Q_{o}^{clim}(x,y,\tau )+Q_{o}^{n\prime }(x,y,\tau ).$$

The superscript *clim* indicates a monthly climatology, which is a sum of all the years for the sorted quantities, and a prime indicates a deviation from this climatology and is the “eddy” terms in this study. For example the SST climatology is quantified as follows:

(A5) $$\theta_{*}^{clim}(x,y,\tau )=\frac{1}{N}\overset{N}{\underset{n=1}{\sum }}\theta_{*}^{n}(x,y,\tau ).$$

The time‐mean SST is written as

(A6) $$\theta_{*}^{m}(x,y)={\bar{\theta }}_{*}(x,y)=\frac{1}{M}\overset{M}{\underset{\tau =1}{\sum }}\theta_{*}^{clim}(x,y,\tau ),$$

where the overbar is a time‐averaging operator and is equivalent to averaging over all months and years,

(A7) $${\bar{\theta }}_{*}(x,y)=\frac{1}{NM}\int_{0}^{t}\theta_{*}(x,y,t)dt^{\prime }=\frac{1}{NM}\overset{M}{\underset{\tau =1}{\sum }}\overset{N}{\underset{n=1}{\sum }}\theta_{*}^{n}(x,y,\tau ).$$

The seasonal climatology anomaly of SST can then be written as

(A8) $$\theta_{*}^{s}(x,y,\tau )=\theta_{*}^{clim}(x,y,\tau )-\theta_{*}^{m}(x,y).$$

The climatology, time‐mean, and seasonal climatology anomaly of net air‐sea heat flux can be derived similarly to SST as in (A5)–(A8); (4) can then be written as

(A9) $$\bar{\theta_{*}^{n}Q_{o}^{n}}=\frac{1}{NM}\overset{N}{\underset{n=1}{\sum }}\overset{M}{\underset{\tau =1}{\sum }}\theta_{*}^{n}Q_{o}^{n}=\theta_{*}^{m}Q_{o}^{m}+\bar{\theta_{*}^{s}Q_{o}^{s}}+\bar{\theta_{*}^{n\prime }Q_{o}^{n\prime }},$$

where cross terms cancel because the sum over years of the “eddy” terms is zero. For example the sum over years of the “eddy” SST (after rearranging (A3)) is

(A10) $$\frac{1}{N}\overset{N}{\underset{n=1}{\sum }}\theta_{*}^{n\prime }(x,y,\tau )=\frac{1}{N}\overset{N}{\underset{n=1}{\sum }}\theta_{*}^{n}(x,y,\tau )-\theta_{*}^{clim}(x,y,\tau )=0,$$

where the first term on the right‐hand side of (A10) is equivalent to the SST climatology (A5). The superscripts *n* in (4) have been dropped for brevity.
